# Supplementary material for: Graphene nanomechanical vibrations measured with a phase-coherent software-defined radio
Source: Commun Eng. 2024 Mar 8;3:45. doi: 10.1038/s44172-024-00186-4 (PMC11101442; doi:10.1038/s44172-024-00186-4)
Supplement: Supplementary file 2 — Supplementary Information [file 44172_2024_186_MOESM2_ESM.pdf]

Supplementary Information for *Graphene nanomechanical vibrations measured with a phase-coherent software-defined radio.*

Ce Zhang[1,2], YuBin Zhang[1,2], Chen Yang[1,2], Heng Lu[1,2], FengNan Chen[1,2], Ying Yan[1,2], and Joel Moser*[1,2]

[1]School of Optoelectronic Science and Engineering & Collaborative Innovation Center of Suzhou Nano Science and Technology, Soochow University, Suzhou, China
[2]Key Lab of Advanced Optical Manufacturing Technologies of Jiangsu Province & Key Lab of Modern Optical Technologies of Education Ministry of China, Soochow University, Suzhou, China

Contents

1 Notation. **2**

2 Estimating the thickness of the resonator. **2**

3 Reflectance and absorbance of few-layer graphene suspended over a reflective substrate. **4**

4 Matlab codes used in this work. **6**

5 Power spectra of displacement fluctuations induced by a force noise. **15**

6 Phase modulation induced by strain modulation. **18**

*j.moser@suda.edu.cn

Supplementary Note 1. Notation.

δz_{m}	complex amplitude of flexural vibrations
ϕ_{m}	phase of vibrations with respect to phase of drive
f_{m}	resonant frequency of vibration mode
k_{m}	spring constant of vibration mode
Q_{m}	quality factor of vibration mode
m_{eff}	effective mass of vibration mode
η	nonlinear damping factor of vibration mode
C_{g}	capacitance between resonator and gate
$V_{\mathrm{g}}^{\mathrm{dc}}$	dc voltage applied between resonator and gate
δV_{d}	rms amplitude of coherent ac voltage applied between resonator and gate
P_{d}	drive power applied to the gate electrode
δF_{d}	rms amplitude of coherent driving force
f_{d}	frequency of coherent driving force
S	single-sided power spectral density
P	power calculated as S multiplied by bandwidth
$\langle\cdot\rangle_m$	average of m realizations
$\mathbb{V}$	variance
$4S_{uu}$	intensity of voltage noise applied to the gate electrode
$h(t) \leftrightarrow \hat{H}(f)$	Fourier transforms in the time and the frequency domains
f	Fourier or spectral frequency
I, Q	in-phase and quadrature components of vibrations
$V_{\mathrm{pd}}(t)$	photodetector output voltage
$\tilde{I}$, $\tilde{Q}$	in-phase and quadrature components of down-converted $V_{\mathrm{pd}}(t)$
κ	spring constant modulation strength
f_{mod}	spring constant modulation frequency
ρ	Fresnel reflection coefficient
d	distance or thickness
λ	wavelength
R	reflectance
A	absorbance

Supplementary Note 2. Estimating the thickness of the resonator.

We estimate the thickness of the resonator using optical reflectometry. Namely, we measure the reflectance of the system composed of few-layer graphene (FLG) in contact with the substrate of silicon oxide-coated silicon (hereafter labelled as R_{FLG}) and the reflectance of the substrate without FLG (hereafter labelled as R_{bare}). We then calculate R_{FLG} and R_{bare} within a model of normally incident plane waves [1]. We find $R_{\mathrm{FLG}} = |c/a|^2$ and $R_{\mathrm{bare}} = |c'/a'|^2$, with

$$\begin{bmatrix} a \\ c \end{bmatrix} = \frac{1}{1+\rho_1}\begin{bmatrix} 1 & \rho_1 \\ \rho_1 & 1 \end{bmatrix}\begin{bmatrix} e^{\mathrm{j}\frac{2\pi}{\lambda}n_{\mathrm{G}}d_{\mathrm{G}}} & 0 \\ 0 & e^{-\mathrm{j}\frac{2\pi}{\lambda}n_{\mathrm{G}}d_{\mathrm{G}}} \end{bmatrix}$$
$$\cdot \frac{1}{1+\rho_2}\begin{bmatrix} 1 & \rho_2 \\ \rho_2 & 1 \end{bmatrix}\begin{bmatrix} e^{\mathrm{j}\frac{2\pi}{\lambda}n_{\mathrm{ox}}d_{\mathrm{ox}}} & 0 \\ 0 & e^{-\mathrm{j}\frac{2\pi}{\lambda}n_{\mathrm{ox}}d_{\mathrm{ox}}} \end{bmatrix}\frac{1}{1+\rho_3}\begin{bmatrix} 1 & \rho_3 \\ \rho_3 & 1 \end{bmatrix}\begin{bmatrix} 1 \\ 0 \end{bmatrix} \quad (1)$$

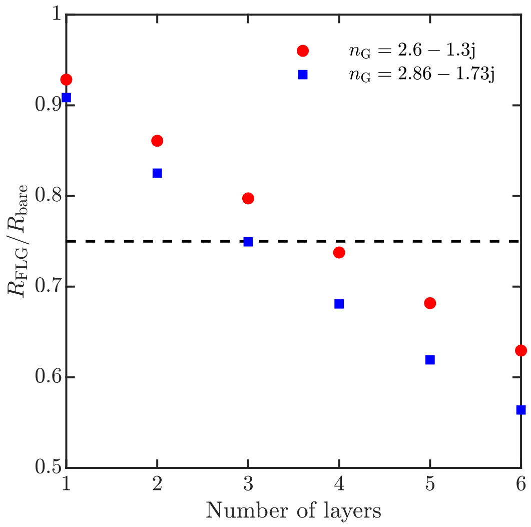

Figure S1: Estimating the thickness of the resonator. Dots: calculated ratio of $R_{\rm FLG}$ (FLG on SiO_2 on Si) to $R_{\rm bare}$ (SiO_2 on Si) at $\lambda = 633$ nm as a function of the number of layers in FLG. Horizontal dashed line shows the measured ratio.

and with

$$\begin{bmatrix} a' \\ c' \end{bmatrix} = \frac{1}{1+\rho_1'} \begin{bmatrix} 1 & \rho_1' \\ \rho_1' & 1 \end{bmatrix} \begin{bmatrix} e^{\mathrm{j}\frac{2\pi}{\lambda} n_{\rm ox} d_{\rm ox}} & 0 \\ 0 & e^{-\mathrm{j}\frac{2\pi}{\lambda} n_{\rm ox} d_{\rm ox}} \end{bmatrix} \frac{1}{1+\rho_3} \begin{bmatrix} 1 & \rho_3 \\ \rho_3 & 1 \end{bmatrix} \begin{bmatrix} 1 \\ 0 \end{bmatrix}, \tag{2}$$

where

$$\begin{aligned} \rho_1 &= \frac{1-n_{\rm G}}{1+n_{\rm G}} \\ \rho_2 &= \frac{n_{\rm G}-n_{\rm ox}}{n_{\rm G}+n_{\rm ox}} \\ \rho_3 &= \frac{n_{\rm ox}-n_{\rm Si}}{n_{\rm ox}+n_{\rm Si}} \\ \rho_1' &= \frac{1-n_{\rm ox}}{1+n_{\rm ox}}\,. \end{aligned}$$

We use the following refractive indices at $\lambda = 633$ nm:

$$\begin{aligned} n_{\rm G} &= 2.6 - \mathrm{j}1.3 \text{ (graphene)} \\ n_{\rm G}' &= 2.86 - \mathrm{j}1.73 \text{ (graphene}') \\ n_{\rm ox} &= 1.476 \ (\mathrm{SiO_2}) \\ n_{\rm Si} &= 3.882 - \mathrm{j}0.0196\,. \end{aligned}$$

The thickness of SiO_2 is $d_{\rm ox} = 500$ nm and the thickness of FLG is $d_{\rm G} = N_{\rm L} \times 0.34$ nm with $N_{\rm L}$ the number of layers. The refractive index for graphene′ is borrowed from Ref. [2]. The refractive index $n_{\rm G}$ used e.g. in Ref [3] yields an absorbance A for a free-standing single layer of graphene *not placed in an optical standing wave* that is close to $\pi\alpha$, where α is the fine structure constant. Indeed, for a single layer we find

$$A = 1 - |c''/a''|^2 - 1/|a''|^2 \simeq \pi\alpha \simeq 0.022\,, \tag{3}$$

with

$$\begin{bmatrix} a'' \\ c'' \end{bmatrix} = \frac{1}{1+\rho_1} \begin{bmatrix} 1 & \rho_1 \\ \rho_1 & 1 \end{bmatrix} \begin{bmatrix} e^{\mathrm{j}\frac{2\pi}{\lambda} n_{\mathrm{G}} d_{\mathrm{G}}} & 0 \\ 0 & e^{-\mathrm{j}\frac{2\pi}{\lambda} n_{\mathrm{G}} d_{\mathrm{G}}} \end{bmatrix} \frac{1}{1-\rho_1} \begin{bmatrix} 1 & -\rho_1 \\ -\rho_1 & 1 \end{bmatrix} \begin{bmatrix} 1 \\ 0 \end{bmatrix} . \tag{4}$$

Figure S1 shows the ratio of calculated R_{FLG} to calculated R_{bare} as a function of the number of graphene layers in the resonator. Both n_{G} and n'_{G} are used. The horizontal dashed line represents the measured ratio. Within our model, we estimate that the resonator is composed of 3 to 4 graphene layers.

Supplementary Note 3. Reflectance and absorbance of few-layer graphene suspended over a reflective substrate.

To optimize the transduction of FLG vibrations into a modulated optical signal, we model our device as a multilayered structure (Fig. S2, top panel) across which normally incident, monochromatic, electromagnetic plane waves propagate. We consider the simplest case of a linear polarization for the real-valued electric field $\mathrm{Re}[\mathbf{E}(z,t)]$, where z is the position along the direction perpendicular to the structure and t is time. Even though incident and reflected waves are circularly polarized between the device and the surface of the quarter-wave plate facing the device, these waves do not traverse any birefringent medium and travel along the same optical path. As a result, incident and reflected waves superimpose themselves on one another and interfere as linearly polarized waves would. The reason for this is that (i) circularly polarized plane waves can be decomposed into two orthogonal, linearly polarized waves; (ii) each of the two incident, linearly polarized waves superimposes on its reflected, linearly polarized wave. We employ a transfer matrix technique to calculate the complex amplitudes E_+, E_- of the forward and backward moving electric field waves (that is, the complex amplitudes of the normal modes of the electromagnetic field) across the structure [4]. With reference to Fig. S2, assuming a light source located away and to the left of FLG, and indexing $E_{q\pm}$ ($E'_{q\pm}$) at the left (right) interface between layer $q-1$ and layer q, we find:

$$\begin{bmatrix} E_{1+} \\ E_{1-} \end{bmatrix} = \mathrm{M}_1\mathrm{P}_1\mathrm{M}_2\mathrm{P}_2\mathrm{M}_3\mathrm{P}_3\mathrm{M}_4\mathrm{P}_4\mathrm{M}_5 \begin{bmatrix} E'_{5+} \\ 0 \end{bmatrix} , \tag{5}$$

with

$$\mathrm{M}_q = \frac{1}{1+\rho_q} \begin{bmatrix} 1 & \rho_q \\ \rho_q & 1 \end{bmatrix} , \rho_q = \frac{n_{q-1}-n_q}{n_{q-1}+n_q}, \mathrm{P}_q = \begin{bmatrix} \exp(\mathrm{j}k_q d_q) & 0 \\ 0 & \exp(-\mathrm{j}k_q d_q) \end{bmatrix} , k_q = \frac{2\pi n_q}{\lambda} , \tag{6}$$

where n_q and d_q are the refractive index and the thickness of layer q, respectively. We use the following refractive indices at $\lambda = 633$ nm:

$$\begin{aligned} n_0 &= 1 \text{ (vacuum)} \\ n_1 &= 2.86 - \mathrm{j}1.73 \text{ (graphene)} \\ n_2 &= 1 \text{ (vacuum)} \\ n_3 &= 0.410 - \mathrm{j}3.498 \text{ (Au)} \\ n_4 &= 1.476 \text{ (SiO}_2\text{)} \\ n_5 &= 3.882 - \mathrm{j}0.0196 \text{ (Si)} . \end{aligned}$$

By propagating $E_\pm(z)$ through the structure and matching them across interfaces, we calculate the total electric field $E(z) = E_+(z) + E_-(z)$ as the superposition of forward and backward moving

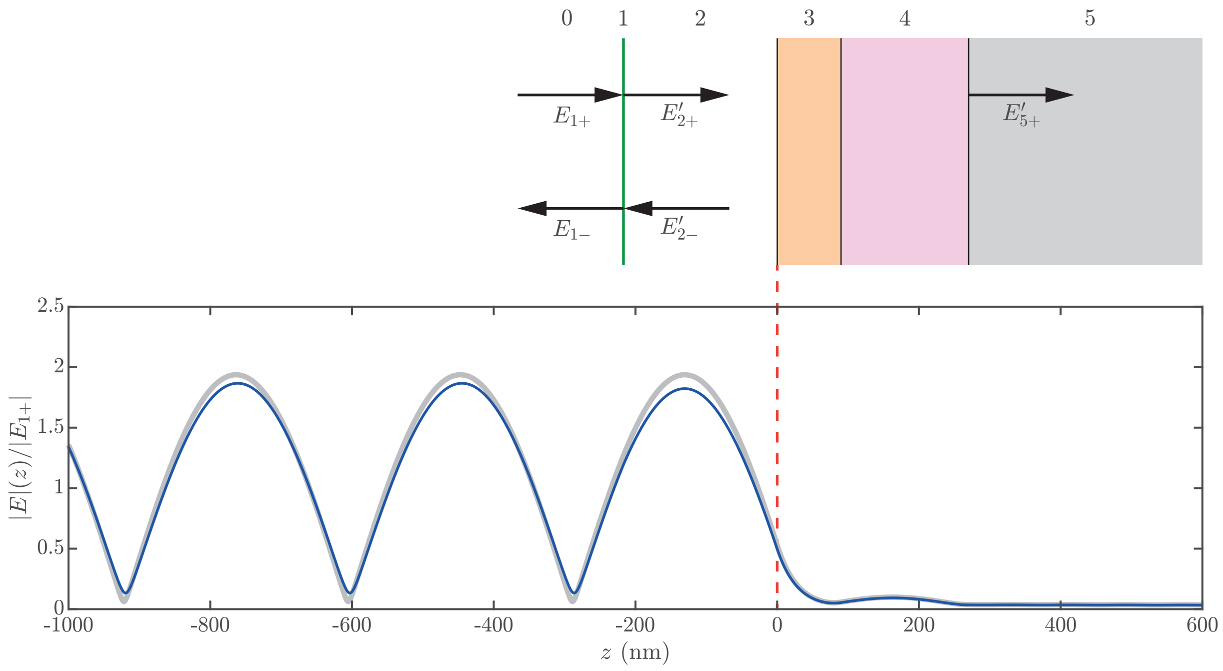

Figure S2: Multilayered structure and optical standing wave. 0: vacuum; 1: FLG; 2: vacuum; 3: Au; 4: SiO_2; 5: Si. $E_{q\pm}$ ($E'_{q\pm}$) are the complex amplitudes of forward $+$ and backward $-$ propagating electric field waves on the left side (on the right side) of the interface between layer $q-1$ and layer q. Arrows indicate the directions of propagation (the field vectors are perpendicular to the directions of propagation and parallel to the interfaces). The plot shows the magnitude of the total electric field $|E(z)| = |E_+(z)+E_-(z)|$ normalized to the magnitude of the incident electric field E_{1+}. Thicker grey trace: no FLG. Thinner blue trace: FLG placed at $|z_{\text{eq}}| = 217$ nm from the surface of the substrate at $z = 0$.

electric field waves. In a steady state, this superposition of propagating fields leads to the buildup of a standing wave, which is located between the device and the surface of the quarter-wave plate facing the device (elsewhere, the linear polarizations of incident and reflected light are orthogonal). The magnitude of this standing wave $|E(z)|$ is shown in Fig. S2, lower panel, both in the case where FLG is absent and in the case where FLG is placed $z_{\text{eq}} = 217$ nm away from the surface of the substrate (see below for the estimation of z_{eq}). FLG mostly acts as an optical absorber (see below) and perturbs the shape of the standing wave only weakly.

Vibrations of FLG about their equilibrium position z_{eq} translate into oscillations in the power of reflected light. We model those vibrations as a modulation of the position of FLG about z_{eq}. Denoting the amplitude of oscillations of reflected optical power by δP_{ref}, the optical power incident on FLG by P_{inc}, the complex reflection coefficient at the interface between vacuum and FLG by $\Gamma_1 = E_{1-}/E_{1+}$, the amplitude of flexural vibrations by δz_{m}, and a fluctuating power background (whose variance depends on P_{inc}) by δP_{bgd}, we write

$$\delta P_{\text{ref}} = P_{\text{inc}} \left.\frac{\mathrm{d}R}{\mathrm{d}z}\right|_{z_{\text{eq}}} \delta z_{\text{m}} + \delta P_{\text{bgd}}\,, \tag{7}$$

with the reflectance $R = |\Gamma_1|^2$. Optimizing the transduction of weak nanomechanical vibrations of amplitude δz_{m} into measurable optical power oscillations δP_{ref} means maximizing $|\mathrm{d}R/\mathrm{d}z|$, which crucially depends on what we call the cavity depth. We define the cavity depth as the nominal

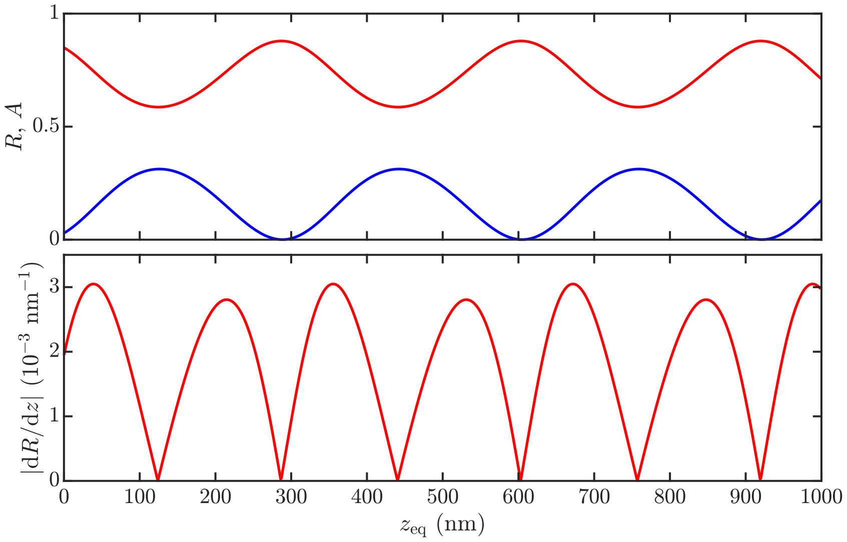

Figure S3: Reflectance R (red), absorbance A (blue) and gradient of reflectance $|\mathrm{d}R/\mathrm{d}z|$ of FLG as a function of distance between FLG and the substrate z_{eq}. Here, FLG is made of 3 layers of graphene. $R + A < 1$ because the substrate is not fully reflective.

distance between a flat FLG and the substrate, that is, the thickness of layer 2 in Fig. S2. We use Eqs. (5), (6) to calculate $|R|$ and $|\mathrm{d}R/\mathrm{d}z|$ as a function of z_{eq} (Fig. S3). We find that $|\mathrm{d}R/\mathrm{d}z|$ exhibits a local maximum near $z_{\mathrm{eq}} \simeq 217$ nm. Because FLG membranes tend to sag ~ 10 nm inside cavities after being transferred onto their subtrate, we design the cavity depth to be a little larger and choose it to be 230 nm. We also calculate the absorbance A of FLG placed in the optical standing wave. It reads [5]:

$$A = (|E_{1+}|^2 + |E'_{2-}|^2 - |E_{1-}|^2 - |E'_{2+}|^2)/|E_{1+}|^2 \,. \tag{8}$$

We display $A(z_{\mathrm{eq}})$ in Fig. S3. The technique of detecting FLG vibrations entirely relies on the fact that graphene is a strong absorber. Indeed, we can verify that if $\mathrm{Im}[n_1] = 0$, then $A = 0$ and $|\mathrm{d}R/\mathrm{d}z| = 0$.

Supplementary Note 4. Matlab codes used in this work.

Code used to compute power estimators. The following is the Matlab code used to compute the three power estimators for the driven response shown in Fig. 2 of the main text.

```
%Frequency sweep parameters
f_start = 70e6;
f_stop = 85e6;
f_step = 100e3;
%Connect to Redpitaya via IP address and port number
IP = '169.254.232.53';
port = 5000;
RP = tcpclient(IP, port);
%Redpitaya parameters
fs = 122.88e6;%sample frequency
```

```
fn = fs/2;%Nyquist frequency
dT = 1/fs;%Sampling time interval
Npoints = 2^14;%Sample points
rbw = fs/Npoints;%Frequency resolution bandwidth
Ntraces = 300;%Number of traces to average
f_trigger = rbw*136;%Trigger frequency (downconvertion frequency)
n = 0:Npoints-1;
f = n*fs/Npoints;%Frequency sequence
%Connect to AWG
AwgAdrress = 'GPIB0::9::INSTR';
AwgObj = visa('agilent',AwgAdrress);
fopen(AwgObj);
%Connect to MXG
MXGobj1 = instrfind('Type', 'gpib', 'BoardIndex', 0, 'PrimaryAddress', ...
    ... 19, 'Tag', '');
if isempty(MXGobj1)
MXGobj1 = gpib('NI', 0, 19);
else
fclose(MXGobj1);
MXGobj1 = MXGobj1(1);
end
fopen(MCGobj1);
%Initialize variables
x=0;
x_num = zeros(1,Npoints);%Data collected in the time domain
x_sum = zeros(1,Npoints);%Sum of the data collected in the time domain
x_avg = zeros(1,Npoints);%Average of the data collected in the time domain
PSDvsum = zeros(1,Npoints);%Sum of the auto power spectrum
PSDdBm = zeros(1,Npoints);%Auto power spectrum
XPSDvsum = zeros(1,Npoints);%Sum of the cross power spectrum
XPSDdBm = zeros(1,Npoints);%Cross power spectrum
peak_auto = zeros(1,N_scan);%Power at f_trigger from atuo power spectrum
peak_cross = zeros(1,N_scan);%Power at f_trigger from cross power spectrum
peak_tavg = zeros(1,N_scan);%Power at f_trigger from auto power spectrum
%of the time averaged signal
f_scan = f_start:f_step:f_stop;%Scanning frequency
N_scan = length(f_scan);
PSDtavg = zeros(1,Npoints);%Auto power spectrum of the time averaged signal
PSDtavgdBm = zeros(1,Npoints);%Auto power spectrum
%of the time averaged signal(in dBm)
figure;
%Start scanning drive frequency
for n_scan = 1:1:N_scan
freqstring_mxg = num2str(f_scan(n_scan));
fprintf(MXGobj1, sprintf('%s','SOURce:FREQuency ',freqstring_mxg));
%set output frequency of MXG
fprintf(AwgObj,'SOURce1:FREQuency %f',f_scan(n_scan)+f_trigger);
%set output frequency of AWG
```

```
pause(1);
%Delay 1s for the resonator to stabilize
k = 0;
for q = 1:1:Ntraces+1
q-1
RP.ByteOrder = 'big-endian';%Set byte order to big-endian
configureTerminator(RP, 'CR/LF');%Configure the terminator as CR/LF
flush(RP);%refresh buffer
writeline(RP,'ACQ:RST');%Reset Sampling
writeline(RP,'ACQ:DEC 1');%Set DEC = 1
writeline(RP,'ACQ:TRIG:LEV 0.0');%Set the trigger level to 0.0
writeline(RP,'ACQ:SOUR1:COUP AC');%Set the AC coupling
writeline(RP,'ACQ:SOUR1:GAIN LV');%Setting the gain to LV
writeline(RP,'ACQ:TRIG:DLY 8192');%Set the trigger delay to 8192
writeline(RP,'ACQ:START');%Start acquisition
pause(0.1);
writeline(RP,'ACQ:TRIG CH2_PE');
%Set the trigger source as positive pulse edge trigger of channel 2
% Waiting for data acquisition to complete
while 1
trig_rsp = writeread(RP,'ACQ:TRIG:STAT?');
if strcmp('TD', trig_rsp(1:2))
break;
end
end
%Acquire the data from Channel 1
x = writeread(RP,'ACQ:SOUR1:DATA?');
x_num = str2num(x(1, 2:length(x)-3));
%Determine if the length of the collection data is correct
if (length(x_num) ~= 2^14)
k = k+1;
clear x_num;
continue;
end
if(q>1)
x_sum = x_sum + x_num;
%Sum the data in time domain
X = fft(x_num,Npoints);
%Fast Fourier transform of the data for this acquisition
Y = fft(y_num,Npoints);
%Fast Fourier transform of the last acquired data
PSDv = X.*conj(X);
%Calculate the auto power spectrum
XPSDv = X.*conj(Y);
%Calculate the cross power spectrum
PSDvsum = PSDvsum + PSDv;
%sum the auto power spectrum
XPSDvsum = XPSDvsum + XPSDv;
```

```
%sum the cross power spectrum
%Averaging the data after sampling Ntraces+1 times
if(q == Ntraces+1)
x_avg = x_sum/(q-k-1);
%Average the data in time domain
X_tavg = fft(x_avg,Npoints);
%Fast Fourier transform of the averaged data
PSDtavg = X_tavg.*X_tavg/Npoints^2;
%Calculate the auto power spectrum
PSDtavgdBm = 10*log10(2*abs(PSDtavg)*1000/50);
%Convert unit to dBm and convert it to single-side auto power spectrum
PSDvsum_temp = PSDvsum/(q-k-1)/Npoints^2;
%Average the auto power spectrum
PSDdBm = 10*log10(abs(PSDvsum_temp)*1000/50) + 10*log10(2);
%Convert unit to dBm and convert it to single-side auto power spectrum
XPSDvsum_temp = XPSDvsum/(q-k-1)/Npoints^2;
%Average the cross power spectrum
XPSDdBm = 10*log10(abs(XPSDvsum_temp)*1000/50) + 10*log10(2);
%Convert unit to dBm and convert it to single-side cross power spectrum
%Save the final power spectrum at drive frequency = f_scan(n_scan)
filename1 = ['F:\ce\dec16\sweep_m50dbm1\PSD_fd',num2str(floor(f_scan(n_scan) ...
    ... /1e6)),'p',num2str(mod(f_scan(n_scan)/1e6*10,10)),'.mat'];
save(filename1,'PSDdBm');
filename2 = ['F:\ce\dec16\sweep_m50dbm1\XPSD_fd',num2str(floor(f_scan(n_scan) ...
    ... /1e6)),'p',num2str(mod(f_scan(n_scan)/1e6*10,10)),'.mat'];
save(filename2,'XPSDdBm');
filename3 = ['F:\ce\dec16\sweep_m50dbm1\PSDtavg_fd',num2str(floor(f_scan(n_scan) ...
    ... /1e6)),'p',num2str(mod(f_scan(n_scan)/1e6*10,10)),'.mat'];
save(filename3,'PSDtavgdBm');
%Extract the power at f_trigger
peak_auto(n_scan) = max(PSDdBm(uint16(Npoints*f_trigger/122.8e6)));
peak_cross(n_scan) = max(XPSDdBm(uint16(Npoints*f_trigger/122.8e6)));
peak_tavg(n_scan) = max(PSDtavgdBm(uint16(Npoints*f_trigger/122.8e6)));
%plot fdrive vs. power response
plot(f_scan(1:n_scan)/1e6,peak_auto(1:n_scan),'r-o');
hold on;
plot(f_scan(1:n_scan)/1e6,peak_cross(1:n_scan),'g-o');
hold on;
plot(f_scan(1:n_scan)/1e6,peak_tavg(1:n_scan),'b-o');
xlabel('f_d [MHz]');
ylabel('XPSD/PSD[dBm]');
legend('Auto PSD','Cross PSD','Average time traces PSD');
end
end
y_num = x_num;
%Record the data from this measurement for the next cycle
%of calculating the cross power spectrum
end
```

```
% Zeroing the variable in preparation for scanning the next drive frequency
x= 0;
x_num = 0;
y_num = 0;
PSDv = 0;
XPSDv = 0;
PSDvsum = zeros(1,Npoints);
PSDdBm = zeros(1,Npoints);
XPSDvsum = zeros(1,Npoints);
XPSDdBm = zeros(1,Npoints);
x_sum = zeros(1,Npoints);
x_avg = zeros(1,Npoints);
PSDtavg = zeros(1,Npoints);
PSDtavgdBm = zeros(1,Npoints);
end
% save the power response at different drive frequency
filename4 = ['F:\ce\dec16\sweep_m50dbm1\PSD_fd_peak','.mat'];
save(filename4,'peak_auto');
filename5 = ['F:\ce\dec16\sweep_m50dbm1\XPSD_fd_peak','.mat'];
save(filename5,'peak_cross');
filename6 = ['F:\ce\dec16\sweep_m50dbm1\PSDtavg_fd_peak','.mat'];
save(filename6,'peak_tavg');
```

Code used to compute averaged cross-spectra. The following is the Matlab code used to compute the cross-power spectra shown in Fig. 3 of the main text and in Fig. S4.

```
% Sampling parameters
recordLen = 1*2^20; %Sample length
Ntrace =1e5; %Average number of times
% Open Scope
scope = visa("ni",'TCPIP0::169.254.200.159::inst0::INSTR');
%Create a communication session with the scope via IP address
set(scope,'InputBufferSize',recordLen*3);
%Allocate buffers to receive data
scope.Timeout = 100;%Set the communication timeout
fopen(scope); %Open the connection to the oscilloscope
% Initialize variables
PSDxbg = zeros(Ntrace,1);
%Auto Power Spectrum Density Background Noise Variable for Channel 1 Signal
PSDybg = zeros(Ntrace,1);
%Auto Power Spectrum Density Background Noise Variable for Channel 2 Signal
XPSDbg = zeros(Ntrace,1);
%Cross Power Spectrum Density Background of the two signals  Noise Variable
Navg = zeros(Ntrace,1);
f1 = figure(1);
f2 = figure(2);
f3 = figure(3);
k = 0;
```

```
p = 0;
for q=1:Ntrace
q
preambleBlock = query(scope,':WAVEFORM:PREAMBLE?');
preambleBlock = regexp(preambleBlock,',','split');
horizlLen = str2double(preambleBlock{3});
%Get number of data points
waveform_XIncrement = str2double(preambleBlock{5});
%Get the increment of the waveform data on the horizontal axis
waveform_YIncrement = str2double(preambleBlock{8});
%Get the increment of the waveform data on the vertical axis
waveform_YOrigin = str2double(preambleBlock{9});
%Get the origin offset of the waveform data on the vertical axis
waveform_Yreference = str2double(preambleBlock{10});
%Get the reference value of the waveform data
recordLen2Transfer = min(recordLen,horizlLen);
%Get the length of the waveform data
fprintf(scope,':RUN');
%Scope starts running and begins to acquire waveform data
pause(0.1);
%Pause for 0.1 seconds to give the scope some time
%to run and prepare the waveform data
fprintf(scope,':STOP'); %Scope stops acquiring waveform data
%Channel 1
fprintf(scope,':WAVEFORM:SOURCE CHANNEL1');
%Set the source of waveform data to channel 1
fprintf(scope,':WAVEFORM:FORMAT WORD');
%Set the format of the waveform data to word(16-bit)
fprintf(scope,':WAVEFORM:BYTEORDER LSBFirst');
%Set the byte order of waveform data to lowest valid byte first
fprintf(scope,':WAVEFORM:STREAMING 1');
%Enable streaming of waveform data
fprintf(scope,':WAVEFORM:DATA?');
%Request waveform data
[waveform_raw,count] = fread(scope,recordLen2Transfer,'int16');
%Read recordLen2Transfer 16-bit integer data from scope and store it
%in waveform_raw array. Count is the number of data successfully read.
x_num =(waveform_raw - waveform_Yreference)*waveform_YIncrement + ...
    ... waveform_YOrigin;
%Process the waveform data and convert it to actual waveform values
%Channel 2
fprintf(scope,':WAVEFORM:SOURCE CHANNEL2');
%Set the source of waveform data to channel 2
fprintf(scope,':WAVEFORM:FORMAT WORD');
%Set the format of the waveform data to word(16-bit)
fprintf(scope,':WAVEFORM:BYTEORDER LSBFirst');
%Setting the byte order of waveform data to lowest valid byte first
fprintf(scope,':WAVEFORM:STREAMING 1');
```

```
%Enable streaming of waveform data
fprintf(scope,':WAVEFORM:DATA?');
%Request waveform data
[waveform_raw2,count] = fread(scope,recordLen2Transfer,'int16');
%Read recordLen2Transfer 16-bit integer data from scope and store it
%in waveform_raw array. Count is the number of data successfully read.
y_num = (waveform_raw2 - waveform_Yreference)*waveform_YIncrement + ...
    ... waveform_YOrigin;
%Process the waveform data and convert it to actual waveform values
t = 0:waveform_XIncrement:(length(x_num)-1)*waveform_XIncrement;
%Get the time vector
%First waveform acquisition
if(q == 1)
Npoints = length(x_num); %Get the length of the data
PSDxsum = zeros(Npoints,1);
%Initialize auto power spectral density of channel 1 data
PSDysum = zeros(Npoints,1);
%Initialize auto power spectral density of channel 2 data
XPSDsum = zeros(Npoints,1);
%Initialize cross power spectral density of channel 1 and channel 2 data
fs = 1/waveform_XIncrement; %Get sample frequency
rbw = fs/Npoints;  %Get resolution bandwidth
n = 0:Npoints-1;
f = n*fs/Npoints;  %Get Frequency sequence
end
%If the data lengths of the two channels are different, interrupt this cycle
%and clear the data of the two channels
if(length(x_num) ~= length(y_num))
k = k+1;
clear x_num;
clear y_num;
continue;
end
X = fft(x_num,Npoints);
%Fast Fourier Transform (FFT) of channel 1 signal x_num(CH1)
Y = fft(y_num,Npoints);
%Fast Fourier Transform (FFT) of channel 2 signal y_num(CH2)
PSDx = X.*conj(X);
%Calculate the auto power spectrum of the signal x_num(CH1)
PSDy = Y.*conj(Y);
%Calculate the auto power spectrum of the signal y_num(CH2)
XPSD = X.*conj(Y);
%Calculate the cross power spectrum of signal x_num(CH1) and signal y_num(CH2)
PSDxsum = PSDxsum + PSDx;
%Sum the auto power spectrum of x_num(CH1)
PSDysum = PSDysum + PSDy;
%Sum the auto power spectrum of y_num(CH2)
XPSDsum = XPSDsum + XPSD;
```

```
%Sum the cross power spectrum
if(q == 1 || q == 10 || mod(q,5) == 0)
PSDxavg_temp = PSDxsum/(q-k)/Npoints^2/rbw;
%Average auto power spectum of x_num(CH1) and convert it to
%single-side auto power spectral density
PSDxdBm = 10*log10(2*abs(PSDxavg_temp)*1000/50);
%Convert unit from V^2/Hz to dBm/Hz
PSDyavg_temp = PSDysum/(q-k)/Npoints^2/rbw;
%Average auto power spectum of y_num(CH2) and convert it to
%single-side auto power spectral density
PSDydBm = 10*log10(2*abs(PSDyavg_temp)*1000/50);
%Convert unit from V^2/Hz to dBm/Hz
XPSDavg_temp = XPSDsum/(q-k)/Npoints^2/rbw;
%Average cross power spectum and convert it to
%single-side cross power spectral density
XPSDdBm = 10*log10(2*abs(XPSDavg_temp)*1000/50);
%Convert unit from V^2/Hz to dBm/Hz
%Record the background noise and the corresponding averaging times
p = p+1;
PSDxbg(p) = 10*log10(2*mean( PSDxavg_temp(uint16(Npoints*55e6/fs): ...
    ... uint16(Npoints*60e6/fs)))*1000/50);
PSDybg(p) = 10*log10(2*mean( PSDyavg_temp(uint16(Npoints*55e6/fs): ...
    ... uint16(Npoints*60e6/fs)))*1000/50);
XPSDbg(p) = 10*log10(2*mean(abs(XPSDavg_temp(uint16(Npoints*55e6/fs): ...
    ... uint16(Npoints*60e6/fs))))*1000/50);
Navg(p) = q;
if(q == 1 || q == 10 || q== 100 || q== 200 || q== 300 || q ==400 || q ==500 ...
    ... || q ==600 || q ==700 || q ==800 || q ==900 || mod(q,1000) == 0)
%Plot the auto power spectral density of CH1 and CH2
clf(f1);
figure(f1);
plot(f(1:Npoints/2)/1e6,PSDxdBm(1:Npoints/2),'r','LineWidth',2);
hold on;
plot(f(1:Npoints/2)/1e6,PSDydBm(1:Npoints/2),'g','LineWidth',2);
xlim([60e6/1e6 90e6/1e6]);
ylim([-120 -65])
xlabel('f [MHz]');
ylabel('XPSD/PSD[dBm/Hz]');
legend('Auto PSD of CH1','Auto PSD of CH2','Location','southwest');
s = sprintf('average times = %d',q);
title(s);
%Plot the cross power spectral density
clf(f2);
figure(f2);
plot(f(1:Npoints/2)/1e6,XPSDdBm(1:Npoints/2),'b','LineWidth',2);
% Plot the spectrum
xlim([60e6/1e6 90e6/1e6]);
ylim([-120 -65])
```

```
xlabel('f [MHz]');
ylabel('XPSD/PSD[dBm/Hz]');
legend('Cross PSD','Location','southwest');
s = sprintf('average times = %d',q);
title(s);
% Plot background noise vs. average number of times
figure(f3);
semilogx(Navg(1:p),PSDxbg(1:p),'r','LineWidth',2);
hold on
semilogx(Navg(1:p),PSDybg(1:p),'g','LineWidth',2);
hold on;
semilogx(Navg(1:p),XPSDbg(1:p),'b','LineWidth',2);
xlabel('N Averages');
ylabel('XPSD/PSD[dBm/Hz]');
legend('Bg of Auto PSD1','Bg of Auto PSD2','Bg of Cross PSD', ...
    ... 'Location','southwest');
%Save auto power spectral density vs frequency
%and cross power spectral density vs frequency
PSDx_nodrive_f = zeros(Npoints,2);
PSDy_nodrive_f = zeros(Npoints,2);
XPSD_nodrive_f = zeros(Npoints,2);
PSDx_nodrive_f(:,1) = f;
PSDx_nodrive_f(:,2) = PSDxdBm;
PSDy_nodrive_f(:,1) = f;
PSDy_nodrive_f(:,2) = PSDydBm;
XPSD_nodrive_f(:,1) = f;
XPSD_nodrive_f(:,2) = XPSDdBm;
filename1 = ['F:\ce\Apr18\100mVrms2\PSDx_nodrive_f_',num2str(q),'avg.txt'];
save(filename1,'-ascii','PSDx_nodrive_f');
filename2 = ['F:\ce\Apr18\100mVrms2\PSDy_nodrive_f_',num2str(q),'avg.txt'];
save(filename2,'-ascii','PSDy_nodrive_f');
filename3 = ['F:\ce\Apr18\100mVrms2\XPSD_nodrive_f_',num2str(q),'avg.txt'];
save(filename3,'-ascii','XPSD_nodrive_f');
%Save background noise vs. average number of times
PSDxbg_Avg = zeros(p,2);
PSDybg_Avg = zeros(p,2);
XPSDbg_Avg = zeros(p,2);
PSDxbg_Avg(:,1) = Navg(1:p);
PSDxbg_Avg(:,2) = PSDxbg(1:p);
PSDybg_Avg(:,1) = Navg(1:p);
PSDybg_Avg(:,2) = PSDybg(1:p);
XPSDbg_Avg(:,1) = Navg(1:p);
XPSDbg_Avg(:,2) = XPSDbg(1:p);
filename4 = ['F:\ce\Apr18\100mVrms2\PSDxbg_Avg.txt'];
save(filename4,'-ascii','PSDxbg_Avg');
filename5 = ['F:\ce\Apr18\100mVrms2\PSDybg_Avg.txt'];
save(filename5,'-ascii','PSDybg_Avg');
filename6 = ['F:\ce\Apr18\100mVrms2\XPSDbg_Avg.txt'];
```

```
save(filename6,'-ascii','XPSDbg_Avg');
pause(1);
end
end
end
```

Supplementary Note 5. Power spectra of displacement fluctuations induced by a force noise.

Figure S4 shows the averaged single-sided cross-power spectral density of voltage fluctuations measured at nodes v_1 and v_2 (see Fig. 1a in the main text) as a function of Fourier frequency. This averaged spectral density is labeled as $|\langle S_{v_2 v_1}\rangle_m|$ in the main text. It results from the average of $m = 10^5$ cross-power spectral density spectra computed from 10^5 v_1 and v_2 time traces. The intensity of voltage noise $4S_{uu}$ applied to the gate electrode increases from (a) through (k) (see captions for details). Red traces are fits to Lorentzian lineshapes. Figure S4(l), bottom right panel, shows the full width at half maximum Δf extracted from the Lorentzian fits as a function of S_{uu}. The black trace is a fit of these data to a nonlinear damping model. It is obtained by numerically solving the following equation of motion:

$$\delta\ddot{z}_{\rm m} + \left[\frac{\omega_{\rm m}}{Q_{\rm m}} + \frac{\eta}{m_{\rm eff}}\delta z_{\rm m}^2(t)\right]\delta\dot{z}_{\rm m} + \omega_{\rm m}^2\delta z_{\rm m}(t) = \frac{\delta F(t)}{m_{\rm eff}}\,, \tag{9}$$

with $\delta z_{\rm m}(t)$ the fluctuating displacement, $\omega_{\rm m} = 2\pi f_{\rm m}$ the angular resonant frequency, $Q_{\rm m}$ the quality factor, η the nonlinear damping coefficient, and $m_{\rm eff}$ the effective mass of the mode. $\delta F(t)$ is a fluctuating force that is delta-correlated and whose single-sided power spectral density reads $S_{FF} = 4S_{uu}(C'_{\rm g}V_{\rm g}^{\rm dc})^2$, with $C'_{\rm g} \simeq 8\times10^{-10}$ F m^{-1} the gradient of gate capacitance simulated with COMSOL using the geometry of the device and $V_{\rm g}^{\rm dc} = -15$ V the dc voltage between the gate and the resonator. The factor of 4 accounts for the full reflection of radio frequency waves at the gate due to impedance mismatch. We find that the model reproduces $\Delta f(S_{uu})$ well using $Q_{\rm m} = 160$ and $\eta = 0.9\times10^{13}$ kg m^{-2} s^{-1}. Please note that, in Fig. S4, S_{uu} is the calibrated power spectral density of voltage fluctuations measured across the 50 Ohm input impedance of a spectrum analyzer.

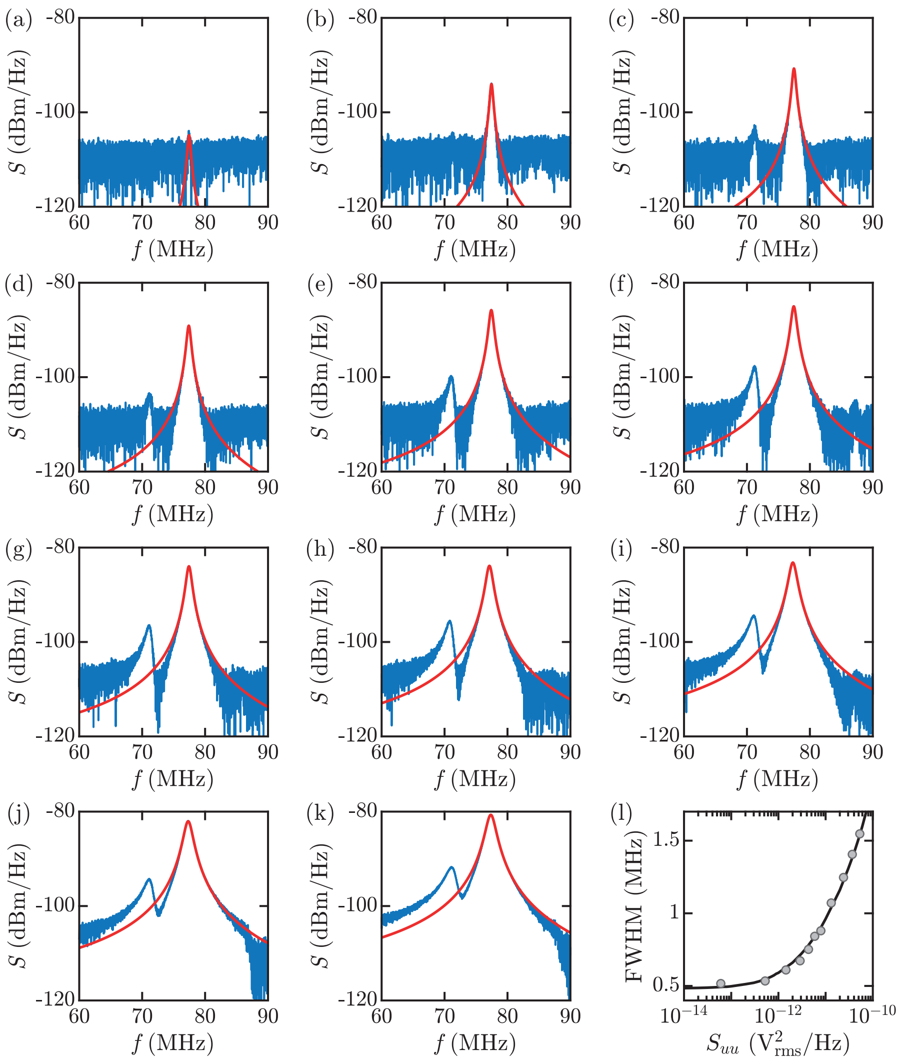

Figure S4: Averaged single-sided cross-power spectral density of voltage fluctuations $|\langle S_{v_2v_1}\rangle_m|$ with $m = 10^5$. (a)-(k): $S_{uu} \simeq 6 \times 10^{-14}$ V^2_{rms} Hz^{-1} (a); $S_{uu} \simeq 5.2 \times 10^{-13}$ V^2_{rms} Hz^{-1} (b); $S_{uu} \simeq 1.4 \times 10^{-12}$ V^2_{rms} Hz^{-1} (c); $S_{uu} \simeq 2.9 \times 10^{-12}$ V^2_{rms} Hz^{-1} (d); $S_{uu} \simeq 4.3 \times 10^{-12}$ V^2_{rms} Hz^{-1} (e); $S_{uu} \simeq 5.9 \times 10^{-12}$ V^2_{rms} Hz^{-1} (f); $S_{uu} \simeq 7.9 \times 10^{-12}$ V^2_{rms} Hz^{-1} (g); $S_{uu} \simeq 1.3 \times 10^{-11}$ V^2_{rms} Hz^{-1} (h); $S_{uu} \simeq 2.4 \times 10^{-11}$ V^2_{rms} Hz^{-1} (i); $S_{uu} \simeq 3.7 \times 10^{-11}$ V^2_{rms} Hz^{-1} (j); $S_{uu} \simeq 5.3 \times 10^{-11}$ V^2_{rms} Hz^{-1} (k). (l) Measured full width at half maximum of the power spectra, $\Delta f(S_{uu})$ (dots), and fit based on Eq. (9).

Figure S5 displays a simulation of Brownian motion based on the geometrical parameters of the resonator in the main text. $\langle S_{v_1v_1}\rangle_m$ (light blue) and $|\langle S_{v_2v_1}\rangle_m|$ (dark blue) are shown as a function of Fourier frequency. From a to f, m ranges from 1 to 10^5. The power spectral density of voltage fluctuations is $S_{uu} = -115$ dBm Hz^{-1} $= 1.58 \times 10^{-13}$ V^2_{rms} Hz^{-1}. The simulation is based on the formalism developed in Ref. [6]. We find that it reproduces the measured spectrum well.

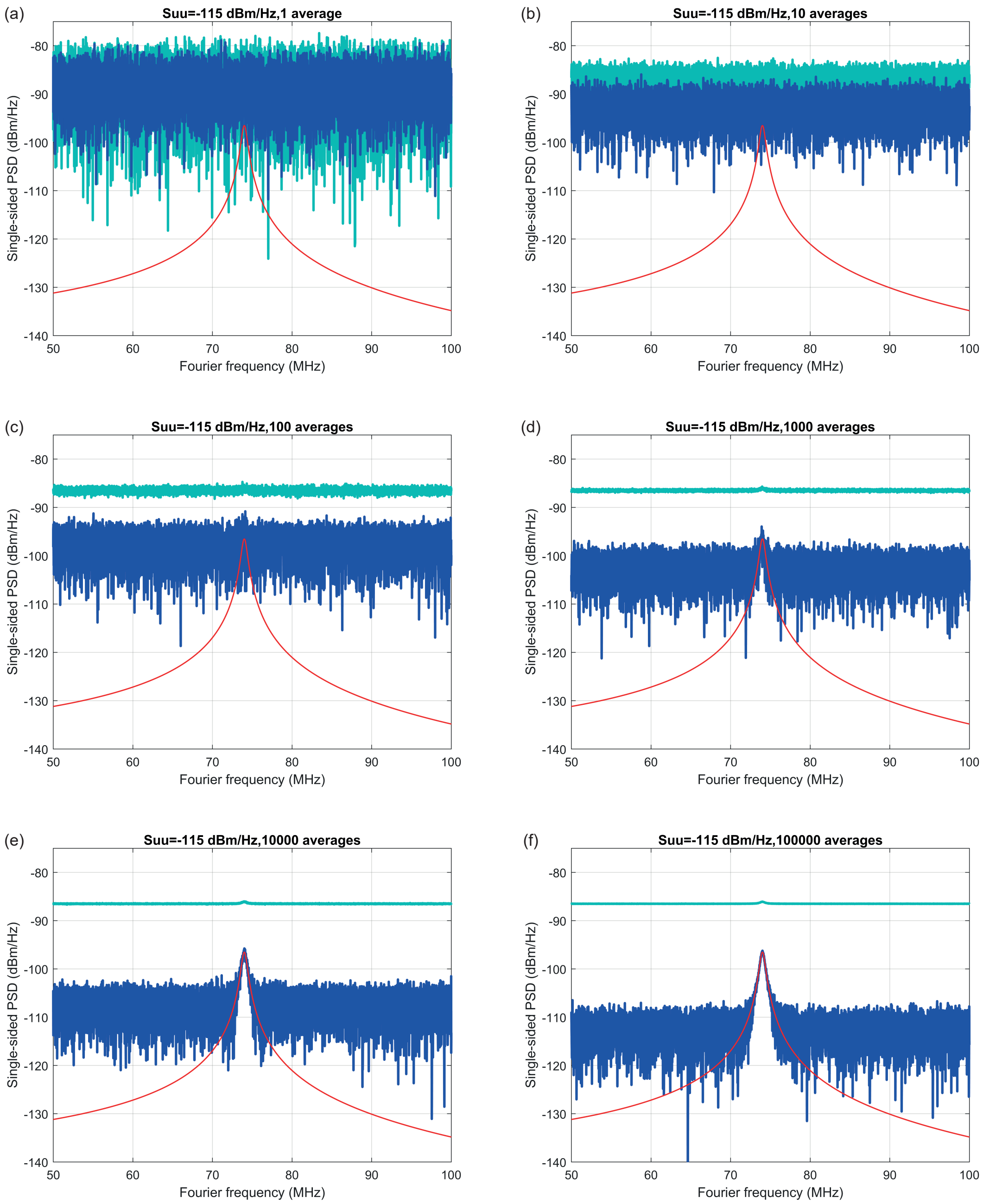

Figure S5: Simulation of the Brownian motion of the resonator. $\langle S_{v_1v_1}\rangle_m$ (light blue) and $|\langle S_{v_2v_1}\rangle_m|$ (dark blue) are shown as a function of Fourier frequency for m ranging from 1 to 10^5. The red trace is the response of a harmonic oscillator based on m_{eff}, f_{m} and Q_{m} of the vibrational mode and on a m^2-to-dBm transduction factor used as a fit parameter.

Supplementary Note 6. Phase modulation induced by strain modulation.

Details of the dynamics of $\tilde{I}$ and $\tilde{Q}$ for various spring constant modulation strengths κ and modulation frequencies f_{mod} are shown in Figs. S6, S7, S8, and S9, where $P_{\text{d}} = -40$ dBm and $V_{\text{g}}^{\text{dc}} = -14.7$ V. Black traces are obtained by solving a linear equation of motion for the resonator that includes a modulated spring constant of the form $k_{\text{m}}[1 - \kappa \cos(2\pi f_{\text{mod}} t)]$. Figure S6 shows $\tilde{I}$, $\tilde{Q}$ and ϕ_{m} as a function of time for $f_{\text{mod}} = 3.8$ MHz and for $\kappa = 0.08$ (a), $\kappa = 0.04$ (b), $\kappa = 0.02$ (c), and $\kappa = 0$ (d). Similarly, Figure S7 is for $f_{\text{mod}} = 7.6$ MHz, Fig. S8 is for $f_{\text{mod}} = 9.5$ MHz, and Fig. S9 is for $f_{\text{mod}} = 15.2$ MHz.

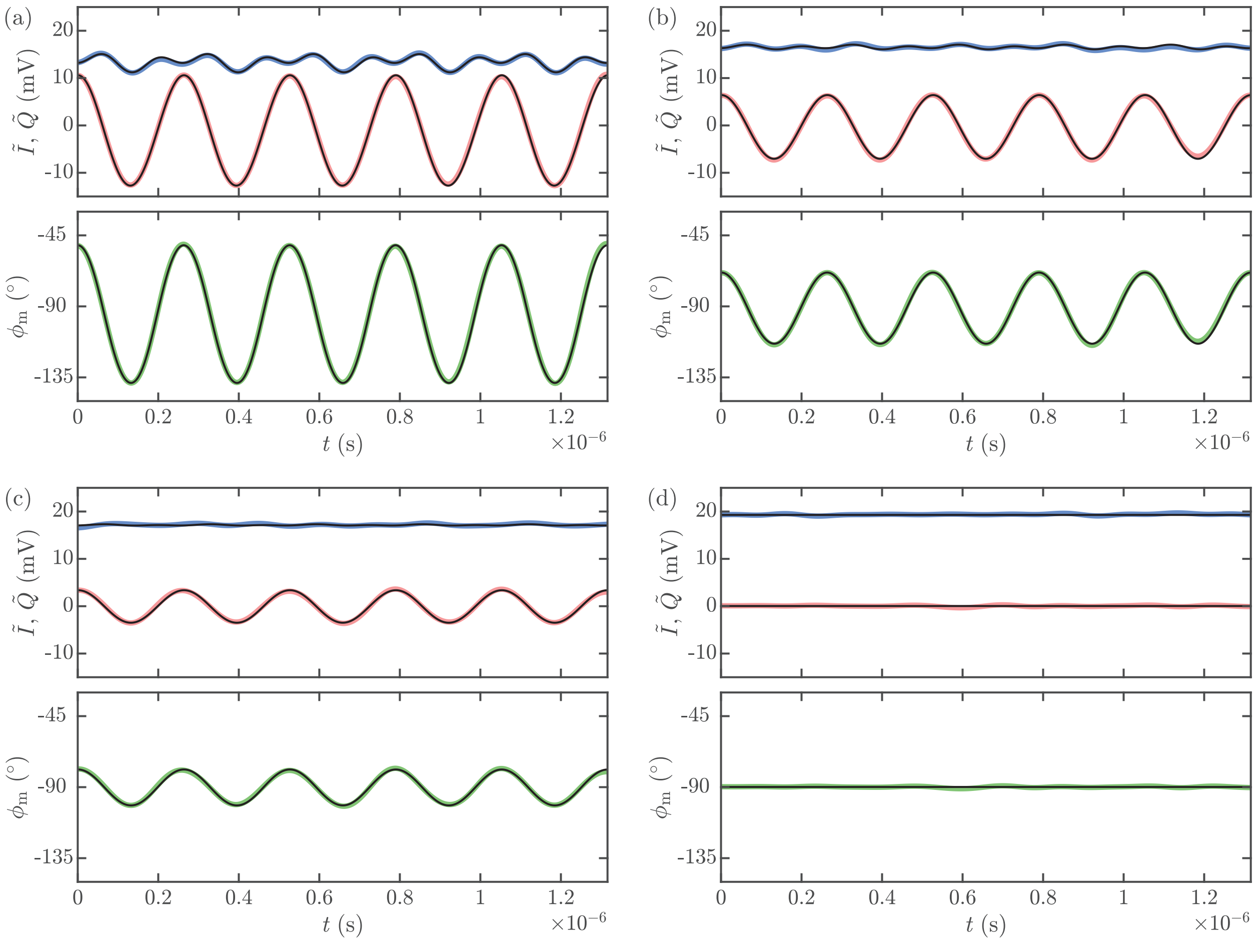

Figure S6: $\tilde{I}$, $\tilde{Q}$ and ϕ_{m} as a function of time for $f_{\text{mod}} = 3.8$ MHz and for $\kappa = 0.08$ (a), $\kappa = 0.04$ (b), $\kappa = 0.02$ (c), and $\kappa = 0$ (d).

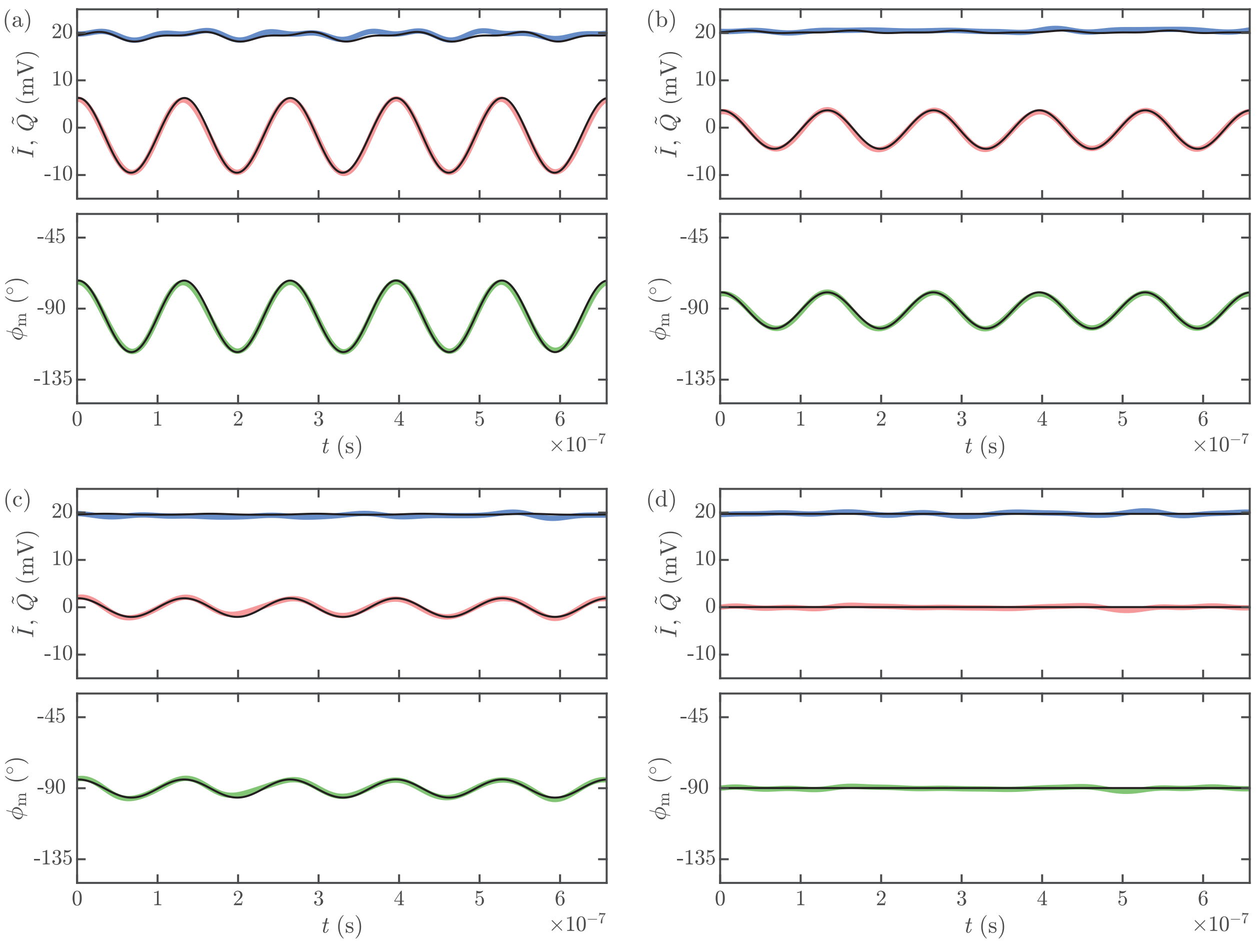

Figure S7: $\tilde{I}$, $\tilde{Q}$ and $\phi_{\rm m}$ as a function of time for $f_{\rm mod} = 7.6$ MHz and for $\kappa = 0.08$ (a), $\kappa = 0.04$ (b), $\kappa = 0.02$ (c), and $\kappa = 0$ (d).

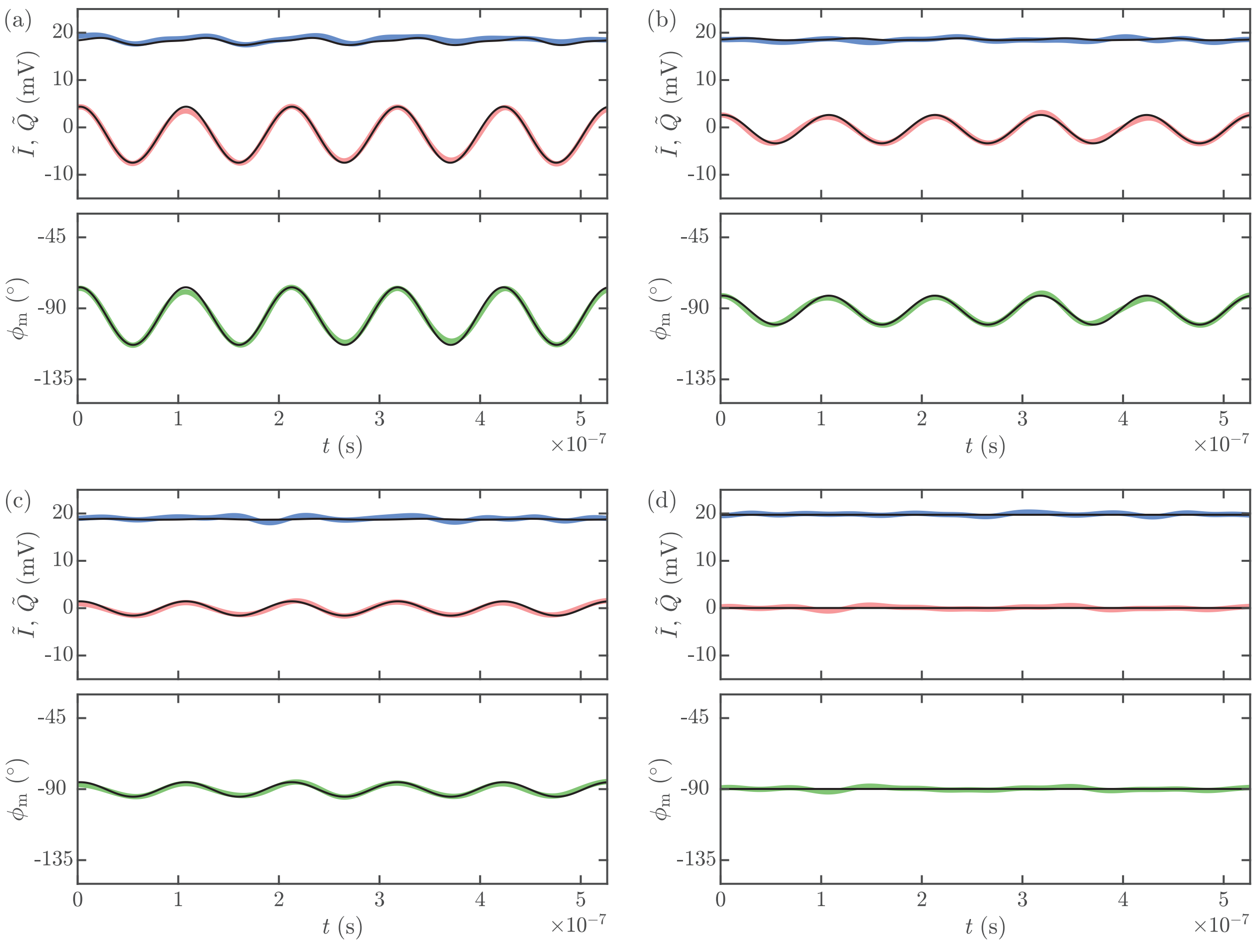

Figure S8: $\tilde{I}$, $\tilde{Q}$ and ϕ_{m} as a function of time for $f_{\mathrm{mod}} = 9.5$ MHz and for $\kappa = 0.08$ (a), $\kappa = 0.04$ (b), $\kappa = 0.02$ (c), and $\kappa = 0$ (d).

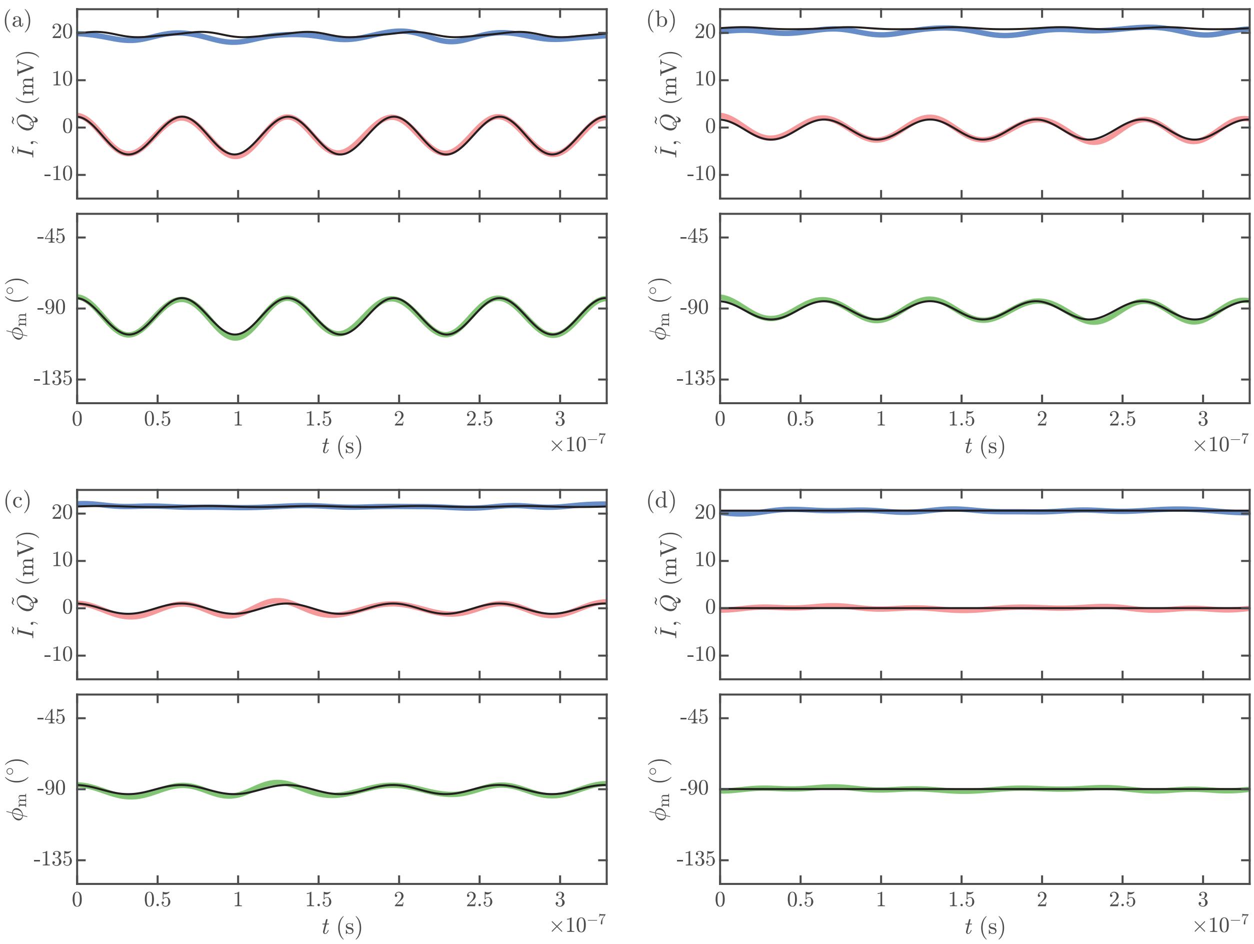

Figure S9: $\tilde{I}$, $\tilde{Q}$ and ϕ_m as a function of time for $f_\mathrm{mod} = 15.2$ MHz and for $\kappa = 0.08$ (a), $\kappa = 0.04$ (b), $\kappa = 0.02$ (c), and $\kappa = 0$ (d).

Figure S10 displays calculations showing the effect of f_mod on the dynamics of the in-phase I and quadrature Q components of vibrations. Parameters are $f_\mathrm{m} = 76$ MHz, $Q_\mathrm{m} = 110$, $m_\mathrm{eff} = 10^{-17}$ kg, $P_\mathrm{d} = -40$ dBm, and $V_\mathrm{g}^\mathrm{dc} = -14.7$ V. Geometrical parameters of the resonator in the main text are used. The spring constant modulation strength is $\kappa = 0.02$. Figure S10a maps I and Q over a short time span for $f_\mathrm{mod} = 10^4$ Hz (black trace). The arc in red shows the extent of vibrational phase modulation amplitude $2\Delta\phi_\mathrm{m}$. The circle in grey maps the in-phase and quadrature components of vibrations without modulation at $\kappa = 0$, $(f_\mathrm{m}^2 - f_\mathrm{d}^2)/D \times \sqrt{2}\delta F_\mathrm{d}/m_\mathrm{eff}$ and $(f_\mathrm{m} f_\mathrm{d}/Q_\mathrm{m})/D \times \sqrt{2}\delta F_\mathrm{d}/m_\mathrm{eff}$, with $D = (f_\mathrm{m}^2 - f_\mathrm{d}^2)^2 + (f_\mathrm{m} f_\mathrm{d}/Q_\mathrm{m})^2$, as f_d is swept through resonance. Figure S10b shows the power spectral density $S_{\phi\phi}$ of ϕ_m as a function of Fourier frequency f normalized to f_mod. At such low f_mod, ϕ_m swings to large values and the phase response is nonlinear. Figures S10c, d show the same analysis for $f_\mathrm{mod} = 10^5$ Hz. Figures S10e, f are for $f_\mathrm{mod} = 10^6$ Hz. Figures S10g, h are for $f_\mathrm{mod} = 2 \times 10^6$ Hz. Figures S10i, j are for $f_\mathrm{mod} = 4 \times 10^6$ Hz.

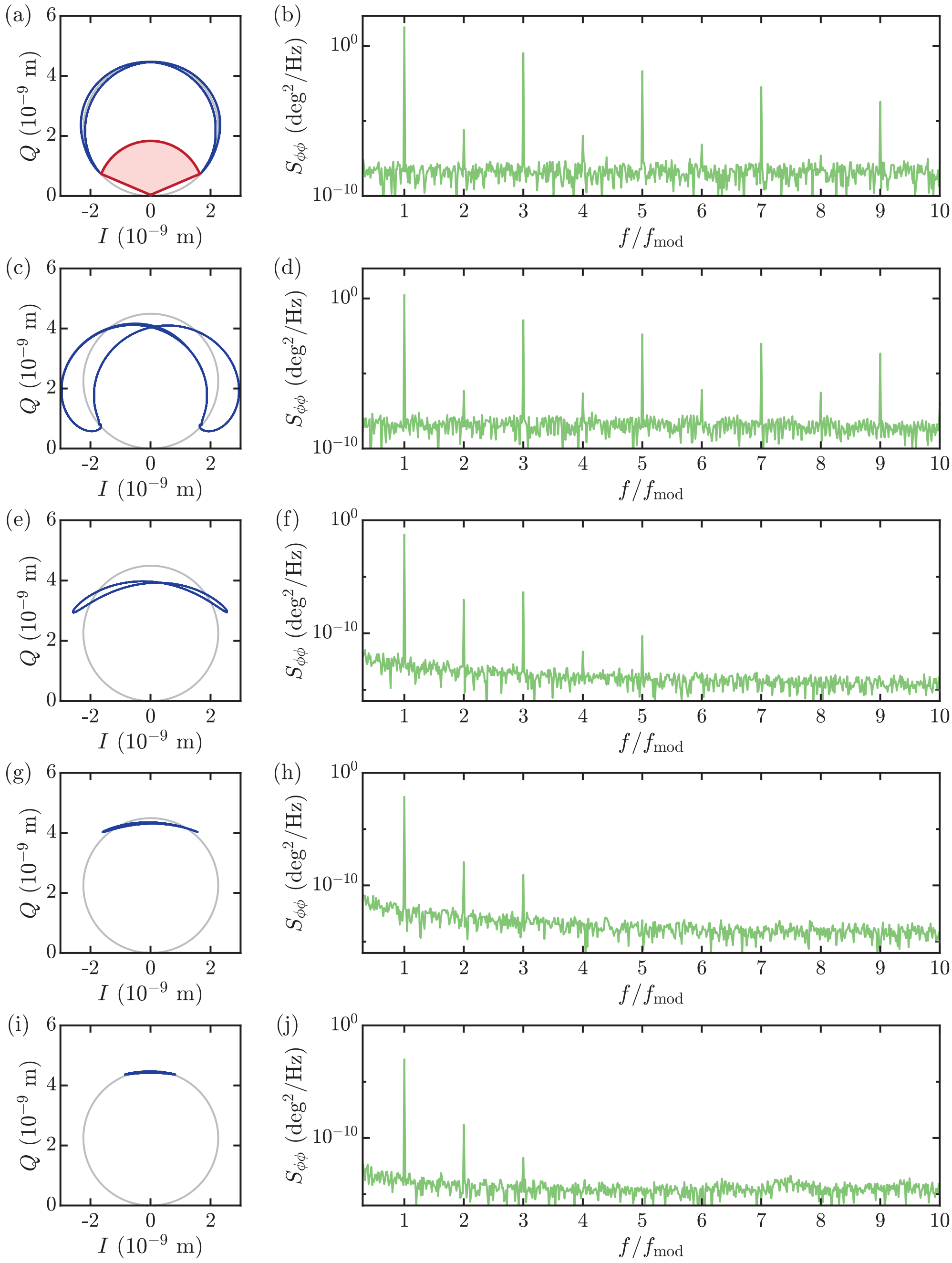

Figure S10: Calculated in-phase I and quadrature Q components of vibrations and power spectral densities of vibrational phase $S_{\phi\phi}$ as a function of Fourier frequency f for modulation strength $\kappa = 0.02$. (a, b) $f_{\mathrm{mod}} = 10^4$ Hz. (c, d) $f_{\mathrm{mod}} = 10^5$ Hz. (e, f) $f_{\mathrm{mod}} = 10^6$ Hz. (g, h) $f_{\mathrm{mod}} = 2\times10^6$ Hz. (i, j) $f_{\mathrm{mod}} = 4 \times 10^6$ Hz.

Supplementary References

[1] Roddaro, S., Pingue, P., Piazza, V., Pellegrini, V. & Beltram, F. The optical visibility of graphene: Interference colors of ultrathin graphite on SiO_2. *Nano Lett.* **7**, no. 9, 2707–2710 (2007).

[2] Wang, Z. & Feng, P.X.-L. Interferometric motion detection in atomic layer 2D nanostructures: visualizing signal transduction efficiency and optimization pathways. *Sci. Rep.* **6**, 28923 (2016).

[3] Storch, I. R., De Alba, R., Adiga, V. P., Abhilash, T. S., Barton, R. A., Craighead, H. G., Parpia, J. M. & McEuen, P. L. Young's modulus and thermal expansion of tensioned graphene membranes. *Phys. Rev. B* **98**, no. 8, Art. no. 085408 (2018).

[4] S. J. Orfanidis. Electromagnetic Waves and Antennas. Available online at http://eceweb1.rutgers.edu/ orfanidi/ewa/ [accessed: Feb. 6, 2024]

[5] F. Chen, C. Yang, W. Mao, H. Lu, K. G Schaedler, A. Reserbat-Plantey, J. Osmond, G. Cao, X. Li, C. Wang, Y. Yan, and J. Moser. Vibration detection schemes based on absorbance tuning in monolayer molybdenum disulfide mechanical resonators. *2D Mater.* **6**(1), 011003 (2018).

[6] Nørrelykke, S. F. & Flyvbjerg, H. Harmonic oscillator in heat bath: Exact simulation of time-lapse-recorded data and exact analytical benchmark statistics. *Phys. Rev. E* **83**, 041103 (2011).
